# Interpreting patient-Specific risk prediction using contextual decomposition of BiLSTMs: application to children with asthma

**DOI:** 10.1186/s12911-019-0951-4

**Published:** 2019-11-08

**Authors:** Rawan AlSaad, Qutaibah Malluhi, Ibrahim Janahi, Sabri Boughorbel

**Affiliations:** 1 Machine Learning Group, Sidra Medicine, Doha, Qatar; 2 Department of Computer Science and Engineering, Qatar University, Doha, Qatar; 3 Division of Pediatric Pulmonology, Sidra Medicine, Doha, Qatar

**Keywords:** Interpretability, Deep learning, Predictive models, Electronic health record

## Abstract

**Background:**

Predictive modeling with longitudinal electronic health record (EHR) data offers great promise for accelerating personalized medicine and better informs clinical decision-making. Recently, deep learning models have achieved state-of-the-art performance for many healthcare prediction tasks. However, deep models lack interpretability, which is integral to successful decision-making and can lead to better patient care. In this paper, we build upon the contextual decomposition (CD) method, an algorithm for producing importance scores from long short-term memory networks (LSTMs). We extend the method to bidirectional LSTMs (BiLSTMs) and use it in the context of predicting future clinical outcomes using patients’ EHR historical visits.

**Methods:**

We use a real EHR dataset comprising 11071 patients, to evaluate and compare CD interpretations from LSTM and BiLSTM models. First, we train LSTM and BiLSTM models for the task of predicting which pre-school children with respiratory system-related complications will have asthma at school-age. After that, we conduct quantitative and qualitative analysis to evaluate the CD interpretations produced by the contextual decomposition of the trained models. In addition, we develop an interactive visualization to demonstrate the utility of CD scores in explaining predicted outcomes.

**Results:**

Our experimental evaluation demonstrate that whenever a clear visit-level pattern exists, the models learn that pattern and the contextual decomposition can appropriately attribute the prediction to the correct pattern. In addition, the results confirm that the CD scores agree to a large extent with the importance scores generated using logistic regression coefficients. Our main insight was that rather than interpreting the attribution of individual visits to the predicted outcome, we could instead attribute a model’s prediction to a group of visits.

**Conclusion:**

We presented a quantitative and qualitative evidence that CD interpretations can explain patient-specific predictions using CD attributions of individual visits or a group of visits.

## Background

The exponential surge in the amount of digital data captured in electronic health record (EHR) offers promising opportunities for predicting the risk of potential diseases and better informs decision-making. Recently, deep learning models have achieved impressive results, compared to traditional machine learning techniques, by effectively learning non-linear interactions between features for several clinical tasks [1–5]. Among a variety of deep learning methods, recurrent neural networks (RNNs) could incorporate the entire EHR to produce predictions for a wide range of clinical tasks [6–11]. Consequently, there is a growing realization that, in addition to predictions, deep learning models are capable of producing knowledge about domain relationships contained in data; often referred to as interpretations [12, 13].

However, the high-dimensionality and sparsity of medical features captured in the EHR makes it more complex for clinicians to interpret the relative impact of features and patterns which are potentially important in decisions. A patient’s EHR usually consists of a sequence of visits a patient has made, and each visit captures the list of diagnosis codes documented by the clinician. Therefore, it is reasonable and important to have interpretable models which can focus on patient visits that have higher impact on the predicted outcome, ignore those visits with little effect on the outcome, and identify and validate the relevant subset of visits driving the predictions.

Interpreting deep models trained on EHR data for healthcare applications is a growing field spanning a range of techniques, which can be broadly categorized into three classes: attention mechanism, knowledge injection via attention, and knowledge distillation [1]. Attention-mechanism-based learning was used in [14–20] for explaining what part of historical information weighs more in predicting future clinical events. Knowledge injection via attention often integrates biomedical ontologies, as a major source of biomedical knowledge, into attention models to enhance interpretability, as demonstrated in [16]. Knowledge distillation first trains a complex, slow, but accurate model and then compresses the learned knowledge into a much simpler, faster, and still accurate model, as shown in [21, 22]. However, the majority of previous work has focused on assigning importance scores to individual features. As a result, these techniques only provide limited local interpretations and do not model fine-grained interactions of groups of input features. In addition, most of these techniques require modifications on standard deep learning architectures to make it more interpretable. By contrast, there are relatively few methods that can extract interactions between features that a deep neural network (DNN) learns. In the case of LSTMs, a recent work by Murdoch et al. [23] introduced contextual decomposition (CD), an algorithm for producing phrase-level importance scores from LSTMs without any modifications to the underlying model, and demonstrated it on the task of sentiment analysis.

In this paper, we hypothesized that the CD interpretability method translates well to healthcare. Therefore, we build upon the CD technique and extend it to BiLSTMs in the context of predicting future clinical outcomes using EHR data. Particularly, we aimed to produce visit-level CD scores explaining why a BiLSTM model produced a certain prediction using patients’ EHR historical visits. Our main insight was that rather than interpreting the attribution of individual visits to the predicted outcome, we could instead attribute BiLSTM’s prediction to a subset of visits. Our main contributions are as follows:

- We introduce a CD-based approach to determine the relative contributions of single visits and a group of visits in explaining the predicted outcome, and subsequently identify the most predictive subset of visits.
- We develop an interactive visualization and demonstrate, using a concrete case study, how CD scores offer an intuitive visit-level interpretation.
- We evaluate and compare CD interpretations from LSTM and BiLSTM models for the task of predicting which pre-school children with respiratory system-related complications will have asthma at school age.
- On a real EHR dataset comprising 11,071 patients having a total of 3318 different diagnosis codes, we present quantitative and qualitative evidence that CD interpretations can explain patient-specific predictions using CD attributions of individual visits or a group of visits.

## Methods

### EHR data description

The EHR data consists of patients’ longitudinal time-ordered visits. Let *P* denote the set of all the patients {*p*_1_,*p*_2_,…,*p*_|*P*|_}, where |*P*| is the number of unique patients in the EHR. For each patient *p*∈*P*, there are *T*_*p*_ time-ordered visits $V_{1}^{(p)}, V_{2}^{(p)}, \ldots,V_{T_{p}}^{(p)}$. We denote *D*={*d*_1_,*d*_2_,…,*d*_|*D*|_} as the set of all the diagnosis codes, and |*D*| represents the number of unique diagnosis codes. Each visit $V_{t}^{(p)}$, where the subscript *t* indexes the time step, includes a subset of diagnosis codes, which is denoted by a vector $x_{t}^{(p)} \in \{0,1\}^{|D|}$. The *i*-th element in $x_{t}^{(p)}$ is 1 if *d*_*i*_ existed in visit $V_{t}^{(p)}$ and 0 otherwise. For notational convenience, we will henceforth drop the superscript (*p*) indexing patients.

### Long short term memory networks

Long short term memory networks (LSTMs) are a special class of recurrent neural networks (RNNs), capable of selectively remembering patterns for long duration of time. They were introduced by Hochreiter and Schmidhuber [24], and were refined and widely used by many people in following work. For predictive modeling using EHR data, LSTMs effectively capture longitudinal observations, encapsulated in a time-stamped sequence of encounters (visits), with varying length and long range dependencies. Given an EHR record of a patient *p*, denoted by $X = {\{x_{t}\}}_{t =1}^{T}$, where *T* is an integer representing the total number of visits for each patient. The LSTM layer takes X as input and generates an estimate output *Y*, by iterating through the following equations at each time step *t*:

1 $$ i_{t}= \sigma (W_{i}x_{t} + U_{i}h_{t-1} + b_{i})  $$

2 $$ f_{t}= \sigma (W_{f}x_{t} + U_{f}h_{t-1} + b_{f})  $$

3 $$ o_{t}= \sigma (W_{o}x_{t} + U_{o}h_{t-1} + b_{o})  $$

4 $$ g_{t}= tanh (W_{g}x_{t} + U_{g}h_{t-1} + b_{g})  $$

5 $$ c_{t}= f_{t} \odot c_{t-1} + i_{t} \odot g_{t}  $$

6 $$ h_{t}= o_{t} \odot tanh(c_{t})  $$

Where *i*,*f*, and *o* are respectively the input gate, forget gate, and output gate, *c*_*t*_ is the cell vector, and *g*_*t*_ is the candidate for cell state at timestamp *t*, *h*_*t*_ is the state vector, *W*_*i*_,*W*_*f*_,*W*_*o*_,*W*_*g*_ represent input-to-hidden weights, *U*_*i*_,*U*_*f*_,*U*_*o*_,*U*_*g*_ represent hidden-to-hidden weights, and *b*_*i*_,*b*_*f*_,*b*_*o*_,*b*_*g*_ are the bias vectors. All the gates have sigmoid activations and cells have tanh activations.

### Bidirectional long short term memory networks

Bidirectional LSTMs [25] make use of both the past and the future contextual information for every time step in the input sequence *X* in order to calculate the output. The structure of an unfolded BiLSTM consists of a forward LSTM layer and a backward LSTM layer. The forward layer outputs a hidden state $\overrightarrow {h}$, which is iteratively calculated using inputs in the forward or positive direction from time *t*=1 to time *T*. The backward layer, on the other hand, outputs a hidden state $\overleftarrow {h}$, calculated from time *t*=*T* to 1, in the backward or negative direction. Both the forward and backward layer outputs are calculated using the standard LSTM updating equations 1- 6, and the final *h*_*t*_ is calculated as:

7 $$ \overrightarrow{h}= \overrightarrow{LSTM}(x_{t})  $$

8 $$ \overleftarrow{h}= \overleftarrow{LSTM}(x_{t})  $$

9 $$ h_{t}= [\overrightarrow{h},\overleftarrow{h}] = BiLSTM (x_{t})  $$

The final layer is a classification layer, which is the same for an LSTM- or BiLSTM-based architecture. The final state *h*_*t*_ is treated as a vector of learned features and used as input to an activation function to return a probability distribution *p* over *C* classes. The probability *p*_*j*_ of predicting class *j* is defined as follows:

10 $$ p_{j}=\frac {exp(W_{j}\cdot h_{t} +b_{j})}{\sum_{i=1}^{C} exp(W_{i}\cdot h_{t} +b_{i})}  $$

where *W* represents the hidden-to-output weights matrix and *W*_*i*_ is the i-th column, *b* is the bias vector of the output layer and *b*_*i*_ is the i-th element.

### Contextual decomposition of BiLSTMs

Murdoch et al.[23] suggested that for LSTM, we can decompose every output value of every neural network component into relevant contributions *β* and an irrelevant contributions *γ* as:

11 $$ Y= \beta + \gamma  $$

We extend the work of Murdoch et al.[23] to BiLSTMs, in the context of patient visit-level decomposition for analyzing patient-specific predictions made by standard BiLSTMs. Given an EHR record of a patient, $X = {\{x_{t}\}}_{t =1}^{T}$, we decompose the output of the network for a particular class into two types of contributions: (1) contributions made solely by an individual visit or group of visits, and (2) contributions resulting from all other visits of the same patient.

Hence, we can decompose *h*_*t*_ in (6) as the sum of two contributions *β* and *γ*. In practice, we only consider the pre-activation and decompose it for BiLSTM as:

12 $$ W_{j} \cdot (\overrightarrow{h},\overleftarrow{h}) + b_{j}= W_{j} \cdot [\overrightarrow{\beta},\overleftarrow{\beta}] + W_{j} \cdot [\overrightarrow{\gamma},\overleftarrow{\gamma}] + b_{j}  $$

Finally, the contribution of a subset of visits with indexes *S* to the final score of class *j* is equal to *W*_*j*_·*β* for LSTM and $W_{j} \cdot [\overrightarrow {\beta },\overleftarrow {\beta }] $ for BiLSTM. We refer to these two scores as the CD attributions for LSTM and BiLSTM throughout the paper.

### Finding Most predictive subset of visits

We introduce a CD-based approach to find the most predictive subset of visits, with respect to a predicted outcome. More specifically, the goal is to find subset of visits *X*_*S*_∈*X*, where *X*_*S*_ consists of the visits with the highest relevant contribution $W_{j} \cdot [\overrightarrow {\beta },\overleftarrow {\beta }] $ presented to the user.

Algorithm 1 describes the exact steps to find the most predictive subset of visits represented by *X*_*S*_ with the highest relative CD attributions. We consider *V* is the list of all patient visits, *W* is the list of all window sizes to analyse, and each *w*∈*W* is an integer setting the size of the window, *s* is an integer setting the size of the step between windows, *m* is the model to be decomposed (LSTM/BiLSTM). In our context, a sliding window is a time window of fixed width *w* that slides across the list of patient visits *V* with step size *s* and returns the list of *CandidateGroups* (subsets of visits) with the specified *w*. For each of these *CandidateGroups*, the algorithm takes the subset of visits and apply contextual decomposition on the specified model *m* to get the relative contribution scores of this subset of visits against the complete list of patient visits. This procedure is applied iteratively for each window size *w*. Finally, the *group* with the highest CD score is assigned to *X*_*S*_.

This approach, while simple, exhaustively evaluates all possible combinations of subsets of consecutive visits, and then finds the best subset. Obviously, the exhaustive search’s computational cost is high. However, since the total number of visits doesn’t exceed tens usually, going through all possible combinations of consecutive visits is still computationally feasible.


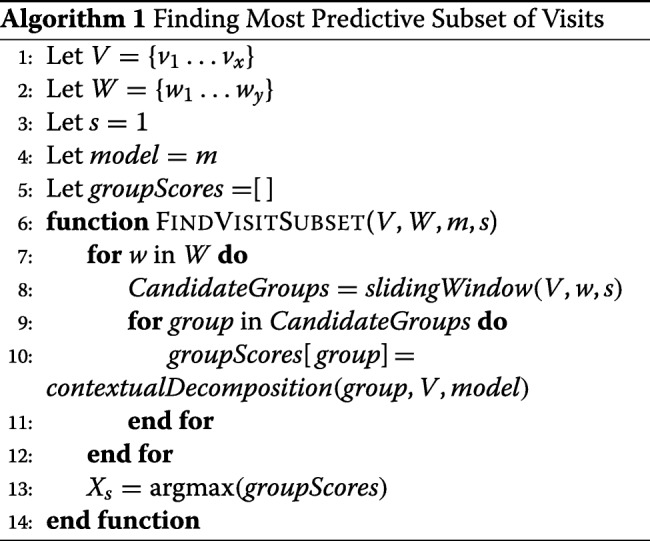


### Dataset and cohort construction

The data was extracted from the Cerner Health Facts^Ⓒ^ EHR database, which consists of patient-level data collected from 561 health care facilities in the United States with 240 million encounters for 43 million unique patients collected between the years 2000-2013 [26]. The data is de-identified and is HIPAA (Health Insurance Portability and Accountability Act)-compliant to protect both patient and organization identity. For the purpose of our analysis, we identified children with respiratory system-related symptoms by following the International Classification of Diseases (ICD-9) standards. We extracted 323,555 children who had a diagnosis code of 786* (symptoms involving respiratory system and other chest symptoms, except 786.3: hemoptysis). After that, we filtered for those patients who had at least one encounter with one of these symptoms and more than two encounters before the age of 5, and were followed-up at least until the age of 8 years. Accordingly, the dataset size reduced significantly to 11,071 patients. The statistics and demographics of the study cohort are described in Table 1.

**Table 1:** Basic statistics of the cohort

		Cases	Controls
# of patients		6159	4912
# of visits		62962	42182
# of diagnosis		128877	77038
Avg. # of visits per patient		10.2	8.6
Avg. # of codes in a visit		2.0	1.8
Gender	Female	2395	2278
	Male	3764	2634
Race	African American	2222	926
	Asian	56	56
	Biracial	83	43
	Caucasian	2361	2805
	Hispanic	602	454
	Native American	22	15
	Pacific Islander	8	2
	Unknown	805	611

To demonstrate our interpretability approach on this data of pre-school children with respiratory system-related symptoms, we try to predict those children who will have asthma at school-age (cases) and those who will not have asthma at school-age (controls). Cases were defined as children who had at least one encounter with respiratory system-related symptoms before the age of 5, and at least one encounter with asthma diagnosis ICD 493* after the age of 6. Controls were defined as children who had at least one encounter with respiratory system-related symptoms before the age of 5, and no diagnosis of asthma for at least three years after school-age, which is age 6. This definition splits our data into 6159 cases and 4912 controls. It is worth mentioning here that, for this specific cohort, the proportion of cases is relatively high (56%), compared to other cohorts or diseases, in which the prevalence of the disease is usually less. The LSTM and BiLSTM models require longitudinal patient-level data that has been collected over time across several clinical encounters. Therefore, we processed the dataset to be in the format of list of lists of lists. The outermost list corresponds to patients, the intermediate list corresponds to the time-ordered visit sequence each patient made, and the innermost list corresponds to the diagnosis codes that were documented within each visit. Only the order of the visits was considered and the timestamp was not included. Furthermore, deep learning libraries assume a vectorized representation of the data for time-series prediction problems. In our case, since the number of visits for each patient is different, we transformed the data such that all patients will have the same sequence length. This is done by padding the sequence of each patient with zeros so that all patients will have the same sequence length, equal to the length of the longest patient sequence. This vectorization allows the implementation to efficiently perform the matrix operations in batch for the deep learning model. This is a standard approach when handling sequential data with different sizes.

### Experimental setup

We implemented LSTM and BiLSTM models in PyTorch, and We also extended the implementation of Murdoch et al.[23] to decompose BiLSTM models. As the primary objective of this paper is not predictive accuracy, we used standard best practices without much tuning to fit the models used to produce interpretations. All models were optimized using Adam [27] with learning rate of 0.0005 using early stopping on the validation set. The total number of input features (diagnosis codes) was 930 for ICD-9 3-digits format and 3318 for ICD-9 4-digits format. Patients were randomly split into training (55%), validation (15%), and test (30%) sets. The same proportion of cases (56%) and controls (44%) was maintained among the training, validation, and test sets. Model accuracy is reported on the test set, and area under the curve (AUC) is used to measure the prediction accuracy, together with 95% confidence interval (CI) as a measure of variability.

## Results

In this section, we first describe the models training results. After that, we provide quantitative evidence of the benefits of using CD interpretations and explore the extent to which it agrees with baseline interpretations. Finally, we present our qualitative analysis including an interactive visualization and demonstrate its utility for explaining predictive models using individual visit scores and relative contributions of subset of visits.

### Models training

To validate the performance of the proposed interpretability approach, we train LSTM and BiLSTM models on the asthma dataset, which has two classes: *c*=1 for cases, and *c*=0 for controls. In addition, we compare the prediction performance of these models with a baseline logistic regression model. The average AUC scores for 10 runs, with random seeds, on the full test set are shown in Table 2. Overall, the LSTM and BiLSTM models achieve higher AUC scores than baseline models such as logistic regression. Consequently, both models learned useful visit patterns for predicting school-age asthma.

**Table 2:** Average AUC of models trained on asthma dataset for the task of school-age asthma prediction

Model	AUC (95% CI)
LSTM	0.831 (0.824-0.838)
BiLSTM	0.819 (0.811-0.827)
Logistic Regression	0.702 (0.692-0.712)

### Quantitative analysis

In this section, we conduct quantitative analysis to (1) validate the contextual decomposition of the trained models, (2) evaluate the interpretations produced by the models, and (3) understand the extent to which the learned patterns correlate with other baseline interpretations.

#### Validation of contextual decomposition for BiLSTMs

**Objective:** To verify that the contextual decomposition of LSTMs and BiLSTMs works correctly with our prediction task, we designed a controlled experiment in which we add the same artificial visit to each patient of certain class, testing whether the contextual decomposition will assign a high attribution score to the artificial visit with respect to that specific class.

Given a patient *p* and a corresponding binary label *c*, we add an artificial visit *v*_*art*_ with one artificial diagnosis code *d*_*art*_ to each patient’s visits list *V*. The *d*_*art*_ was chosen to be a synthetic diagnosis code which does not exist in the ICD-9 codes list. On the full dataset *P*, the artificial visit is added with probability *p*_*art*_ to patients with label 1, and with probability 1−*p*_*art*_ to patients with label 0. As a result, when *p*_*art*_ = 1, all patients of class 1 will have *v*_*art*_, and consequently the model should predict label 1 with a 100% accuracy and contribution of *v*_*art*_ should always be the maximum among other visits. Similarly, when *p*_*art*_ = 0.5, both classes will equally have patients with *v*_*art*_, and therefore *v*_*art*_ does not provide any additional information about the label, and *v*_*art*_ should thus have a small contribution.

**Experimental settings:** We train LSTM and BiLSTM models on the asthma dataset with the artificial visit *v*_*art*_ setup. To measure the impact of *v*_*art*_, we first add *v*_*art*_ to patients of class *c*=1, with probability *p*_*art*_, varying *p*_*art*_ from 1 to 0.5 with steps of 0.1. After that, we train both models on this modified dataset, and then calculate the contribution of each visit by using the CD algorithm. We run the experiment 5 times with a different random seed and report on the average correct attribution. The attribution is correct if the highest contribution among all visits is assigned to *v*_*art*_.

**Fig. 1:**
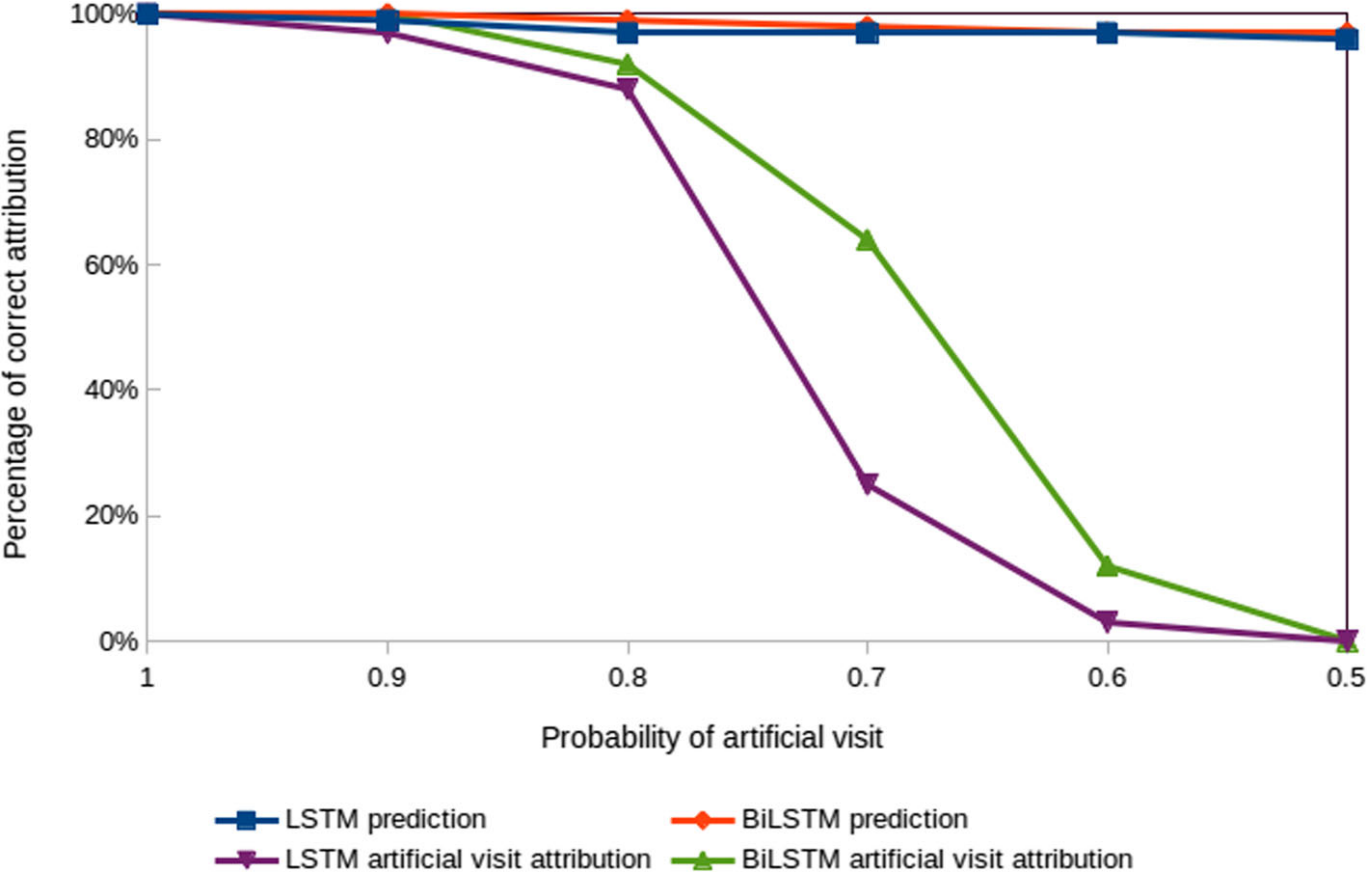
Validation of contextual decomposition for LSTM and BiLSTM for the class *c*=1. The attribution is correct if the highest contribution among all visits is assigned to the artificial visit. The prediction curves indicate the prediction accuracy for class *c*=1, which also represents the upper bound for the attribution accuracy

**Results:** The results of our evaluation are depicted in Fig. 1. When *p*_*art*_ = 1, the models correctly attribute the prediction to the artificial visit at 100% accuracy. Moreover, as *p*_*art*_ becomes smaller, the contribution of the artificial visit goes down, since *v*_*art*_ becomes less important. Finally, when *p*_*art*_= 0.5, the contribution of the artificial visit becomes irrelevant and the model attributes the prediction to other visits. Both models LSTM and BiLSTM perform similarly with 100% and 0% attribution accuracy at *p*_*art*_= 1 and *p*_*art*_=0.5, respectively. However, when *p*_*art*_ is between 0.8 and 0.6, BiLSTM attributes higher contribution to *v*_*art*_ than LSTM. This might be due to BiLSTM specific architecture, which accesses information in both forward and backward direction, allowing it to generate better inference about the visits importance with lower sensitivity to the position of *v*_*art*_, compared to unidirectional LSTM. Overall, we can conclude that whenever there is a clear visit-level pattern, the models learn that pattern and the contextual decomposition can appropriately attribute the prediction to the correct visit.

#### Evaluation of interpretations extracted from BiLSTMs

Before examining the visit-level dynamics produced by the CD algorithm, we first verify that it compares favorably to prior work for the standard use case of producing coefficients for individual visits, using logistic regression. For longitudinal data such as EHR, a logistic regression model summarizes the EHR sequence ensemble to become aggregate features that ignore the temporal relationships among the feature elements. However, when sufficiently accurate in terms of prediction, logistic regression coefficients are generally treated as a gold standard for interpretability. Additionally, when the coefficients are transformed by an exponential function, they can be interpreted as odds ratio [28]. In particular, when applied to clinical outcomes prediction, the ordering of visits given by their coefficient value provides qualitatively sensible measure of importance. Therefore, when validating the interpretations extracted using the CD algorithm we should expect to find a meaningful correlation between the CD scores and the logistic regression coefficients. To that end, we present our evaluation of the interpretations extracted using the CD algorithm with respect to the coefficients produced by logistic regression.

**Generating Ground Truth Attribution for Interpretation:** Using our trained logistic regression model, we identified the most important three visits for each patient and used it as a baseline to evaluate the correlation between logistic regression coefficients and CD attributions. First, we calculated the importance score for each diagnosis code. After that we used these scores to calculate the importance score for each visit, by summing the importance scores of the diagnosis codes included in each visit. The importance score for each diagnosis code is calculated as follows:

- extract statistically significant diagnosis codes, using p-value criterion *p*≤0.05
- for all significant diagnosis codes, calculate coefficients and odds ratios
- filter for diagnosis codes with odds ratio >1
- sort filtered diagnosis codes in descending order according to their odds ratios
- group the sorted diagnosis codes into 4 groups. Diagnosis codes with similar/closer odds ratios are grouped together
- assign an importance score for each group in descending order, based on the odds ratios of diagnosis codes in each group

Finally, we calculated the importance score for each visit, by summing the importance scores of the diagnosis codes occurred in that visit, and used the visits scores to identify the most important three visits for each patient. We run this analysis on a subset of 5000 patients, who have asthma, and for each patient the ground truth attribution baseline is the most important three visits, ordered according to their importance scores.

**Evaluation:** For each patient/ground-truth pair, we measured if the ground truth visits match the visit with the highest CD score for the same patient. We ranked the CD scores of visits for each patient and reported on the matching accuracy between the visit with the highest CD contribution and the three ground truth visits for each patient.

**Fig. 2:**
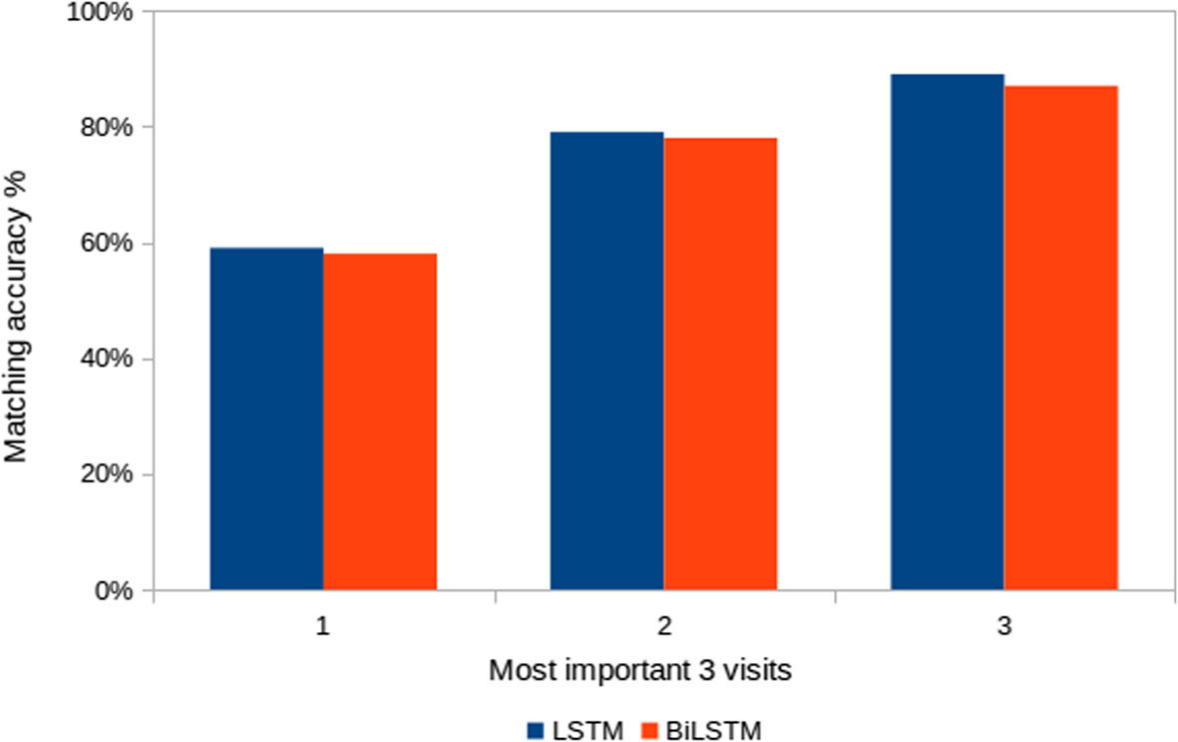
Evaluation of the agreement between CD scores and importance scores generated from logistic regression coefficients. The matching is correct if the visit with the highest LSTM/BiLSTM CD attribution matches one of the top three visits, which are generated using logistic regression coefficients

**Results:** The aggregated results for both LSTM and BiLSTM models are presented in Fig. 2. Overall, we observe that, for the two models, the contextual decomposition attribution overlaps with our generated baseline ground truth attribution for at least 60% of the patient/ground-truth pairs. The matching between the top visit using the CD algorithm and the first top ground truth visit is 60%, the top two ground truth visits is 80%, the top three ground truth visits is 90%. These results confirm that there is a strong relationship between the importance scores generated using logistic regression coefficients and the CD importance scores based on the patterns an LSTM/BiLSTM model learns.

### Qualitative analysis

After providing quantitative evidence of the benefits of CD to interpret the patient EHR visits importance, we now present our qualitative analysis using three types of experiments. First, we introduce our visualization and demonstrate its utility to interpret patient-specific predictions. Second, we provide examples for using our CD-based algorithm to find the most predictive subset of visits. Finally, we show that the CD algorithm is capable of identifying the top scoring visit patterns and demonstrate this in the context of predicting school-age asthma.

#### Explaining predictions using individual visit scores

**Fig. 3:**
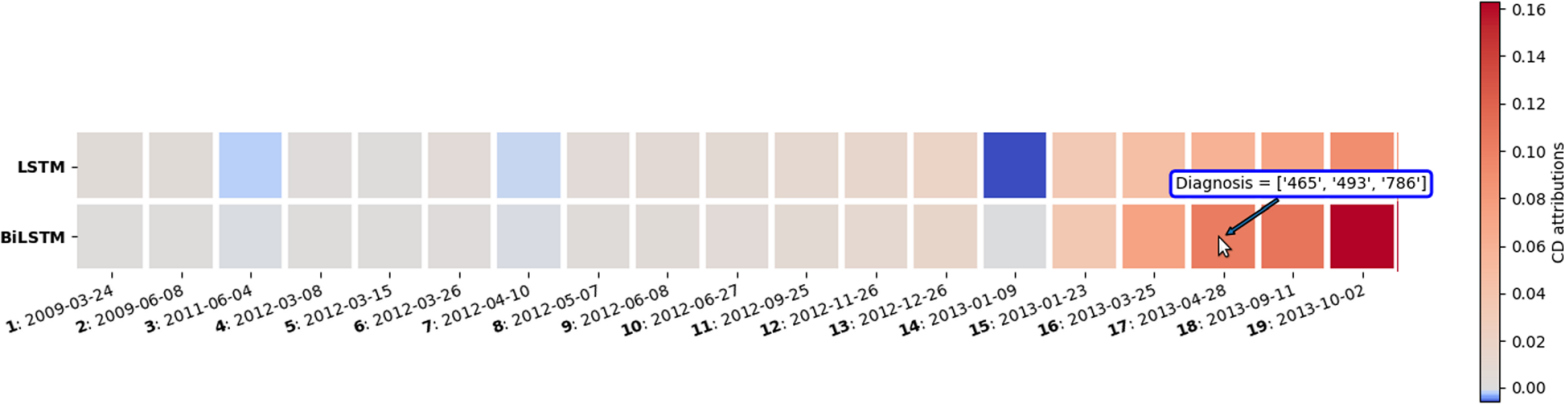
CD scores for individual visits produced from LSTM and BiLSTM models trained for the task of predicting school-age asthma. Red is positive, white is neutral and blue is negative. The squares represent patient EHR time-ordered visits, and the label of each square indicates the visit number appended by the date of the visit. The upper row is the LSTM CD attributions and the lower row is the BiLSTM CD attributions

In this section, we present our interactive visualization and illustrate it with an example for both LSTM and BiLSTM models. The timeline in Fig. 3 represents a patient’s EHR time-ordered visits and the colors of the visits reflect the CD contributions of each visit to the predicted outcome. Moreover, hovering over the visits with the mouse will display the ICD codes documented by the clinician during the visit. Visualizing the CD contributions of each visit can be used to quickly explain why did the model make a certain prediction. For example, the patient shown in Fig. 3 was correctly predicted to have asthma at school age. He had 19 data points (visits) before the age of six years and it was all considered by the model. The visualization indicated that visits 15 to 19 have the highest contribution to the prediction for both LSTM and BiLSTM models, and the ICD-9 codes included in these four visits are: 486 (pneumonia), 786 (symptoms involving respiratory system and other chest symptoms), 493 (asthma), and 465 (acute upper respiratory infections of multiple or unspecified sites). Presenting such information to the clinician could be of a great help in the decision making process. For example, this specific patient has been following up at the hospital from age 0 to 5 years, and he had respiratory-related complications throughout the 5 years. Typically, the physician will have to check the full history of a patient to understand the patient condition and make a decision. In contrast, visualizing the CD scores for each visit as shown in Fig. 3 indicates that, for this specific patient, older visits are not very relevant. The visualization highlights that recent visits are more important to examine. This is probably due to the fact that continuing to have respiratory complications till age 5, just before school-age, is an important indication that this patient will likely continue to have asthma at school age.

#### Explaining predictions using relative contributions of subset of visits

In this section, we first present our results for the implementation of the algorithm introduced earlier for finding the most predictive subset of visits, and then we qualitatively compare between the relative contributions of the subset of visits produced by LSTM and BiLSTM.

**Fig. 4:**
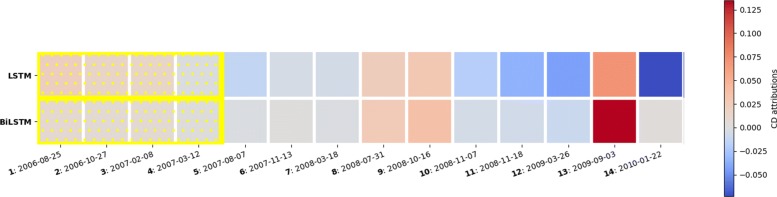
Most predictive subset of visits using CD-based scores highlighted in yellow. Example for a patient where relative contributions of subset of visits produced from LSTM and BiLSTM are similar

Figure 4 shows an example of a patient who was correctly predicted to have asthma at school-age. The patient made 14 visits between age 0 and 5 with different complications. The individual visit scores do not provide clear information about the critical time window which the physician needs to examine. However, using our algorithm for finding the most predictive subset of visits, the algorithm identified that grouping visits 1 to 4 together (highlighted in yellow) produced the maximum relative contribution to the predicted outcome, compared to other subset of visits. The ICD codes included in these visits indicated that this patient has been diagnosed with congenital anomalies as well as asthma before the age of 1, followed by organic sleep disorders and symptoms involving respiratory system and chest in the following years. Therefore, although the contributions of individual visits were not high, the relative contribution of grouping the visits together provided useful information to explain the prediction.

**Fig. 5:**
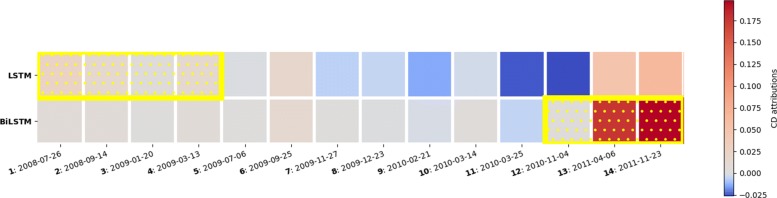
Most predictive subset of visits using CD-based scores. Example for a patient where BiLSTM is producing better interpretation than LSTM

In general, we found that the relative contributions of subset of visits extracted from BiLSTM and LSTM are often similar. However, for some cases, such as the patient shown in Fig. 5, we observed that contributions produced from BiLSMT are likely more clinically relevant than LSTM. This is possibly because BiLSTM mimics physician practice by examining the EHR clinical visits not only in forward time order, but also considers the backward time order so that recent clinical visits are likely to receive higher importance.

#### Identifying top scoring patterns

We now demonstrate the utility of using the CD attributions to identify the top scoring patterns which was learned by the LSTM and BiLSTM models. To address this, we analysed for each patient for which the class *c*=1 (having asthma at school age) was correctly predicted, which visit patterns of length one and two visits had the highest positive contribution towards predicting that class. The results of this evaluation are summarized for one visit patterns in Table 3 and two visits patterns in Table 4. Overall, both models learn similar patterns for both length one and two visits with no significant difference. Moreover, the identified patterns are inline with the risk factors suggested in the literature for school-age asthma [29–31].

**Table 3:** Top scoring patterns of length 1 visit, produced by the contextual decomposition of LSTM and BiLSTM models on the asthma data

	**LSTM**		**BiLSTM**	
	ICD Codes	Frequency%	ICD Codes	Frequency%
1	493.9 Asthma Unspecified	40%	493.9 Asthma Unspecified	34%
2	493.9,786.0 Asthma Unspecified, Dyspnea and Respiratory Abnormalities	13%	786.2 Cough	15%
3	786.0 Dyspnea and Respiratory Abnormalities	11%	493.9,786.0 Asthma Unspecified, Dyspnea and Respiratory	21%
4	493.9,786.2 Asthma Unspecified,Cough	10%	786.0 Dyspnea and Respiratory Abnormalities	10%
5	465.9,493.9 Acute Upper Respiratory Infections of Unspecified Site, Asthma Unspecified	9%	493.9,786.2 Asthma Unspecified, Cough	9%
6	493.0 Extrinsic Asthma	4%	465.9,493.9 Acute Upper Respiratory Infections of Unspecified Site,Asthma Unspecified 8%	
7	486,493.9 Pneumonia, Asthma Unspecified	4%	465.9,786.2 Acute Upper Respiratory Infections of Unspecified Site,Cough	5%
8	465.9,493.9,786.2 Acute Upper Respiratory Infections of Unspecified Site, Asthma Unspecified, Cough	3%	486,493.9 Pneumonia, Asthma Unspecified	3%
9	382.9,493.9 Unspecified Otitis Media, Asthma Unspecified	3%	486,493.9 Pneumonia, Asthma Unspecified	3%
10	493.0, 493.9 Extrinsic Asthma,Asthma Unspecified	3%	V67.9 Unspecified Follow-Up Examination	3%

**Table 4:** Top scoring patterns of length 2 visit, produced by the contextual decomposition of LSTM and BiLSTM models on the asthma data

	**LSTM**		**BiLSTM**	
	ICD Codes	Frequency%	ICD Codes	Frequency%
1	[493.9],[493.9] [Asthma Unspecified],[Asthma Unspecified]	13%	[493.9], [493.9] [Asthma Unspecified],[Asthma Unspecified]	11%
2	[493.9,786.0],[493.9][Asthma Unspecified, Dyspnea and Respiratory Ab-normalities], [Asthma Unspecified]	2%	[493.9,786.0],[493.9][Asthma Unspecified, Dyspnea and Respiratory Ab-normalities], [Asthma Unspecified]	2%
3	[493.9],[493.9,786.0] [Asthma Unspecified], [Asthma Unspecified, Dysp-nea and Respiratory Abnormalities]	2%	[493.9],[493.9,786.0][Asthma Unspecified], [Asthma Unspecified, Dysp-nea and Respiratory Abnormalities]	2%
4	[493.9], [V20.2] [Asthma Unspecified], [Routine Infant or Child Health Check]	2%	[493.9], [V20.2][Asthma Unspecified], [Routine Infant or Child Health Check]	2%
5	[493.9,786.2], [493.9] [Asthma Unspecified, Cough], [Asthma Unspecified]	2%	[493.9,786.2], [493.9][Asthma Unspecified, Cough], [Asthma Unspecified]	1%

## Discussion

In this study, we assessed the potential application of contextual decomposition (CD) method to explain patient-specific risk predictions using quantitative and qualitative evaluation. Our results demonestrated that whenever a clear visit-level pattern exists, the LSTM and BiLSTM models learn that pattern and the contextual decomposition can appropriately attribute the prediction to the correct pattern. In addition, the results confirm that the CD score agrees to a large extent with the importance scores produced using logistic regression coefficients. Our main insight was that rather than interpreting the attribution of individual patient visits to the predicted outcome, we could instead attribute a model’s prediction to a group of visits.

A potential limitation of our study is the identification of asthma patients using ICD codes. In particular, although using ICD codes to identify asthma is a popular practice in large-scale epidemiologic research, previous research showed that using ICD-9 codes have a moderate accuracy of identifying children with asthma, compared to criteria-based medical record review [32]. In addition, the contextual decomposition approach was demonstrated on a single cohort of patients. Generalizing the findings and explanations of this study would require assessing multiple datasets represeting multiple cohorts, diseases, and age groups.

## Conclusion

In this paper, we have proposed using contextual decomposition (CD) to produce importance scores for individual visits and relative importance scores for a group of visits, to explain decisions of risk prediction models. In addition, we developed an interactive visualization tool and demonstrated, using a concrete case study with real EHR data, how CD scores offer an intuitive visit-level interpretation. This movement beyond single visit importance is critical for understanding a model as complex and highly non-linear as BiLSTM. The potential extension of our approach to other sources of big medical data (e.g. genomics and imaging), could generate valuable insights to assist decision-making for improved diagnosis and treatment.

## Data Availability

The data that support the findings of this study are available from Cerner HealthFacts but restrictions apply to the availability of these data, which were used under license for the current study, and so are not publicly available. Data however can be directly requested from Cerner HealthFacts on reasonable request.

## Abbreviations

AUC: Area under the curve
BiLSTM: Bidirectional long short-term memory network
CD: Contextual decomposition
DNN: Deep neural network
EHR: Electronic health record
ICD: International Classification of Diseases
LSTM: Long short-term memory network
RNN: Recurrent neural network
